# Lethal and sub-lethal effects of the insecticide fipronil on juvenile brown shrimp *Farfantepenaeus aztecus*

**DOI:** 10.1038/s41598-018-29104-3

**Published:** 2018-07-17

**Authors:** Ali Abdulameer Al-Badran, Masami Fujiwara, Delbert M. Gatlin, Miguel A. Mora

**Affiliations:** Department of Wildlife and Fisheries Sciences, Texas A&M University, College Station, TX 77843-2258 USA

## Abstract

Chemical pesticides are widely used around the world, but at the same time, they may cause direct or indirect risks to many non-target organisms. Recent increased use of insecticides in coastal areas, for example to control invasive tawny crazy ants, raises concern that insecticides may affect ecologically and/or commercially important species found in estuaries. Here, we investigated the lethal and sub-lethal effects of fipronil on juvenile brown shrimp *Farfantepenaeus aztecus* over 29 days at five different nominal concentrations (0.1, 1.0, 3.0, 6.4, and 10.0 µg/L) in a laboratory experiment. Exposure to all of the fipronil treatments resulted in all individuals dying before the end of the experiment; whereas, no individual died in the control (0.0 µg/L). The 96-hour LC_50_ was determined to be 1.3 µg/L. Shrimp also experienced weight loss under all of the fipronil treatments. Inter-moult interval was increased from 12.2 ± 1.64 day in the control group to 15.5 ± 0.53 day in the 1.0 μg/L treatment. Lipid content of shrimp increased significantly in a concentration-dependent manner. Finally, behavioral and body color changes were also observed under the fipronil treatments. We conclude *F*. *aztecus* is very sensitive to fipronil and monitoring is needed in coastal areas.

## Introduction

Chemical pesticides are commonly used for both agricultural and household purposes world- wide to control pests. However, they are known to have negative side effects on non-target organisms, including terrestrial organisms such as birds^1–3^ and insects^4–6^ as well as aquatic organisms such as fish^7–9^ and arthropods^10,11^. A rapid increase in pesticides use in recent years has resulted in enormous pressure on the ecosystems^12,13^. Pesticides are expected to have a much higher effect on the aquatic environments compared with terrestrial environments, because water bodies are the eventual recipients of these chemicals^14^. The adverse effects of chemical pesticides may be lethal (acute) or sub-lethal (chronic), and the effects can vary depending on species^15^. However, the majority of ecotoxicological studies have focused on the investigation of their lethal effects, neglecting sub-lethal effects. These studies also focus on a few selected model organisms, neglecting effects on other non-target organisms, which may play an important role for ecosystem functions and/or are important for commercial purposes^16–18^.

**Figure 1:**
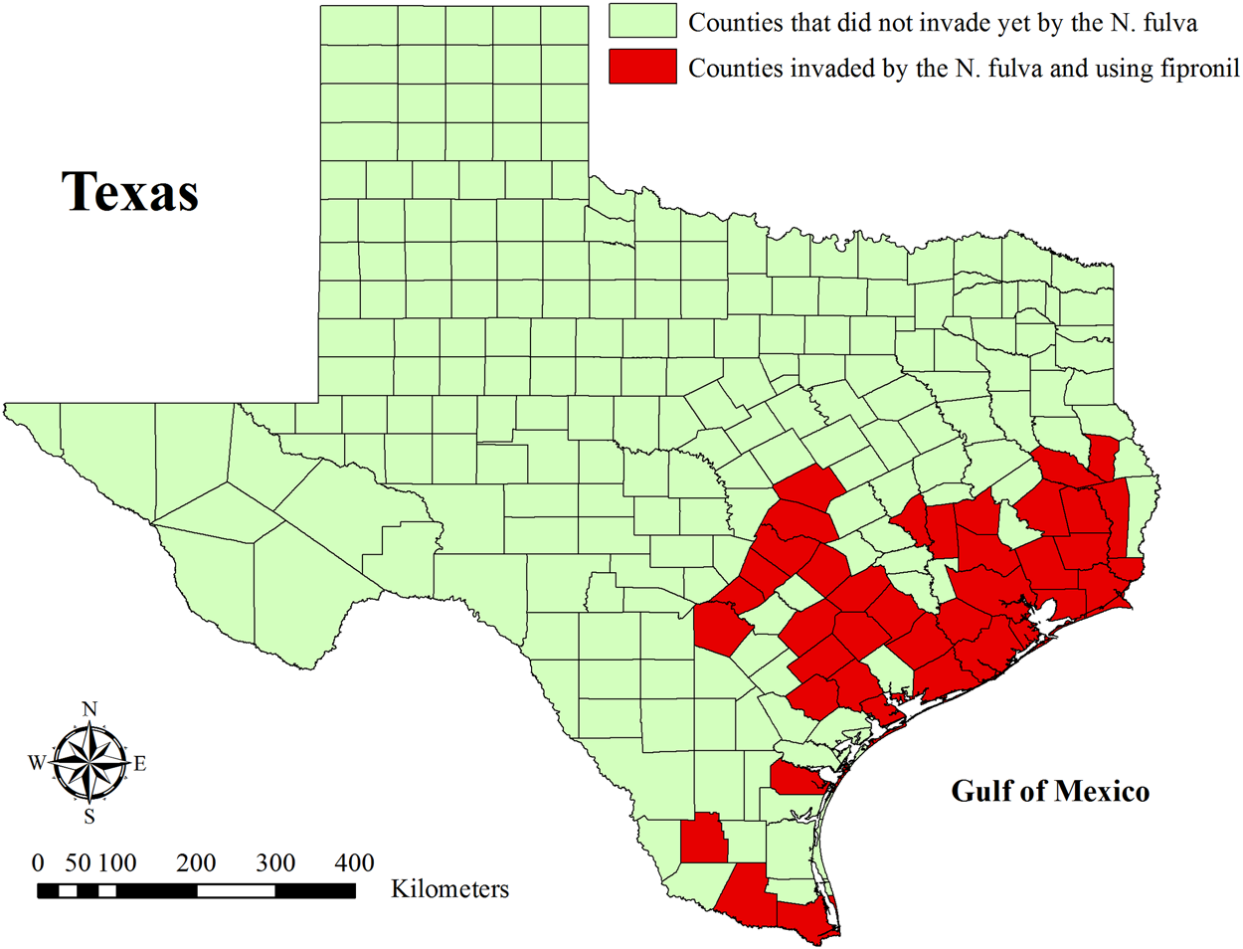
Map of Texas counties. Red color indicate the counties that have been invaded by the tawny crazy ants *N*. *fulva* and that are using fipronil based on the exemption issued by the U.S. EPA in 2016 to control the invasion.

Currently, fipronil is considered one of the most effective phenylpyrazole insecticides, which are used widely, and it is considered to affect arthropods selectively^19,20^. It is used increasingly for the protection of crops such as rice, corn, cotton, potatoes, turnips, and rutabagas from herbivorous insects and for controlling ticks and fleas on animals^13,21,22^. In particular, the use of fipronil has increased in the U.S.A. in recent years in many different states such as California, Louisiana, South Carolina, and Texas. For example, the United States Environmental Protection Agency (U.S. EPA) issued a quarantine exemption to the Texas Department of Agriculture in 2016, allowing the expanded use of fipronil in southeastern counties of Texas to control tawny crazy ants *Nylanderia fulva* (Fig. 1)^23^.

Fipronil can flow into creeks, rivers, and estuaries because it is mobile in soils and soluble in water^24^. Many recent studies have demonstrated the occurrence of fipronil and its degradation products, which have the same or greater toxic properties and are more stable than fipronil itself^21,25–27^, in the aquatic environment at levels ranging between 0.001–10.004 µg/L, often exceeding the acute level (0.1 µg/L) of fipronil in the aquatic life benchmark of the U.S. EPA^22,28–31^. A nationwide survey from 2002 to 2011 conducted by Stone *et al*.^32^ found that fipronil concentrations exceeded its chronic aquatic life benchmark concentration (0.01 µg/L) in about 70% of 125 monitored streams sometime during the survey.

Several studies tested the toxicity of fipronil on non-target aquatic crustaceans such as blue crab *Callinectes sapidus*^33^, Chinese mitten crab *Eriocheir sinensis* and giant river prawn *Macrobranchium rosenbergii*^34^, red swamp crayfish *Procambarus clarkia*^35,36^, white river crayfish *Procambarus zonangulus*^35^, grass shrimp *Palaemonetes pugio*^37,38^, water flea *Daphnia pulex*^39^, and estuarine mysid shrimp *Americamysis bahia*^24^. However, the number of species studied is still limited, and most focused on lethal effects.

The aim of this study was to investigate both lethal and sub-lethal effects of fipronil on the brown shrimp *Farfantepenaeus aztecus*. *F*. *aztecus* is one of the most important commercial fishery species in the U.S., found along the Atlantic coast of the southeastern United States and in the Gulf of Mexico (GOM)^40,41^ with a commercial landing value of $166,542 million in 2016^42^. They are especially abundant along the coasts of Texas and Louisiana, U.S.A. In addition to their economic importance, brown shrimp play an important ecological role for supporting other species^41,43,44^. They are estuarine-dependent during a juvenile stage^40,41^; this potentially exposes them to pesticides that are used on land, because their residues end up in the runoff. The effects of fipronil on penaeid shrimp such as brown shrimp are particularly a concern because of its increased use in coastal communities. In this study, we estimated the effects of fipronil on survivorship, weight gain, inter-moult interval, behavioral changes, and body chemical composition under different nominal concentrations in controlled conditions. The concentrations were selected based on those previously reported for the aquatic environment. We also determined the nominal median lethal concentration (LC_50_) and the nominal median lethal time (LT_50_) of fipronil. These results will fill our knowledge gap in potential effects of fipronil on estuarine crustacean.

## Results

### Water quality

Mean values of water quality parameters were the following: temperature, 20.84 ± 0.24 °C; salinity, 16.20 ± 0.10‰; pH, 8.69 ± 0.15; and dissolved oxygen (DO), 5.67 ± 0.24 mg/L (Table 1**)**. There were no significant differences among treatments for all water quality parameters measured during the experiment, which lasted 29 days, and all of them were within appropriate ranges of the environmental requirements of shrimp^45^.

**Table 1:** Water quality parameters of the shrimp aquariums during 29 days of laboratory experiments.

Fipronil concentrations (µg/L)	Water quality parameters			
	Temp. °C	Salinity ‰	pH	DO mg/L
Control	21.11 ± 0.32^a^	16.11 ± 0.40	8.41 ± 0.16	5.43 ± 0.11
0.1	21.0 ± 0.33	16.16 ± 0.44	8.61 ± 0.12	5.43 ± 0.17
1.0	20.98 ± 0.25	16.23 ± 0.38	8.74 ± 0.03	5.60 ± 0.26
3.0	20.7 ± 0.10	16.30 ± 0.04	8.78 ± 0.02	5.72 ± 0.11
6.4	20.45 ± 0.49	16.34 ± 0.24	8.81 ± 0.02	5.85 ± 0.04
10.0^b^	20.8	16.09	8.81	6.04

^a^Values of Mean ± standard deviation for each parameter of all concentrations (treatments) of fipronil used during the trials.

^b^Values of fipronil concentration 10.0 µg/L have no standard deviation due to shrimp mortality during first few days of the trial.

### Survival, median lethal time (LT_50_), and acute toxicity test (LC_50_)

**Figure 2:**
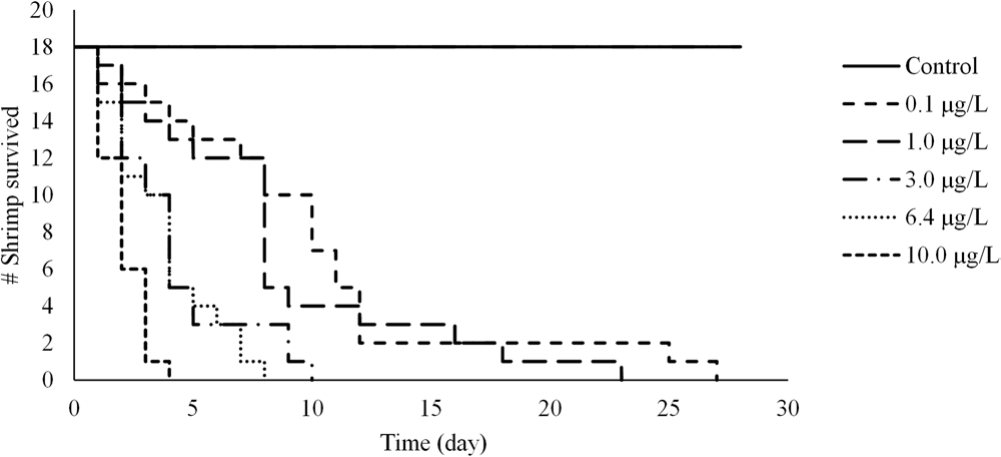
Kaplan-Meier survivorship curves of juvenile shrimp under different concentrations of fipronil during 29 days of exposure. Day 1 is 24-h after the beginning of the experiment. All treatments were significantly different from the control according to the non-parametric Log-Rank test (P < 0.0001).

Our results showed that survival of juvenile shrimp decreased significantly with fipronil concentration, from 0.1 µg/L to 10.0 µg/L (Kaplan-Meier survival curve analysis followed by the non-parametric Log-Rank test, P < 0.0001) as shown in Table 2 and Fig. 2. Starting from week 1, significant differences were detected between a control treatment (with survival rate of 100%) and all other fipronil treatments except 0.1 µg/L treatment, which showed a survival rate of 72.2% over the week. After week 1, all treatments were significantly different from the control (Kaplan-Meier survival curve analysis followed by the non-parametric Log-Rank test, P < 0.0001).

Under the higher concentrations of fipronil (6.4 µg/L and 10 µg/L), shrimp showed faster reduction in survivorship where all individuals died by day 8 and day 4, respectively. Under lower fipronil concentrations (0.1 µg/Land 1.0 µg/L), survivorship declined with time at a slower rate and all individuals died by day 28 and 23, respectively. Under all concentrations of fipronil, the survival rate of juvenile shrimp over the duration of the experiment was 0.0% (Kaplan-Meier survival curve analysis followed by the non-parametric Log-Rank test, P < 0.0001). In comparison, none of the shrimp died in any replicate under the control treatment (0.0 µg/L).

The median lethal time LT_50_ (the time required for 50% of the animals to die at a particular exposure concentration, and also called median time to death) of juvenile shrimp under fipronil treatments ranged between 1.66 ± 0.57 day in the 10.0 µg/L treatment to 6.66 ± 3.51 day in the 0.1 µg/L treatment. One-way Analysis of Variance ANOVA (P < 0.05) showed that all treatments were significantly different from the control which showed no mortality among the shrimp during the experiment (Table 2). Fipronil 96-h LC_50_ (lethal concentration to reach 50% mortality within 96 hours) of juvenile shrimp was 1.3 µg/L with the 95% confidence interval ranging from (1.0 to 1.5). Table 3 compares the results from this study with those from previous studies obtained by others.

**Table 2:** Weekly survival rate (mean ± standard deviation) of juvenile shrimp starting from day1 to the end of the experiment.

Fipronil concentrations (µg/L)	n	LT_50_ (day)	Survival %				
			Day 1	Week 1	Week 2	Week 3	Week 4
Control	18	/	100	100	100	100	100
0.1	18	6.66* ± 3.51	100	72.20 ± 25.46	16.63* ± 16.65	11.10* ± 19.22	0
1.0	18	6.33* ± 3.78	100	66.63* ± 28.86	16.63* ± 16.65	5.53* ± 9.58	0
3.0	18	2.66* ± 1.15	100	16.63* ± 16.65	0	0	0
6.4	18	3.0* ± 1.0	100	16.60 *	0	0	0
10.0	18	1.66* ± 0.57	100	0	0	0	0

n = number of shrimp individuals in each treatment (6 shrimp per replicate aquarium, 3 aquariums per treatment). LT_50_ is the time required for 50% of shrimp to die after the exposure to fipronil under each treatment, measured per day in this study. Values with star (*) indicate treatment is significantly different from the control (P < 0.05).

**Table 3:** Median lethal concentration (LC_50_) for *F*. *aztecus* and other estuarine and freshwater arthropods (Crustacean species) exposed to fipronil for 96-h in toxicity tests.

Species	Common name	Habitat	LC_50_ (µg/L)	Reference
*Americamysis bahia*	Mysid shrimp	Estuarine	0.14	^24^
*Palaemonetes pugio*	Grass shrimp	Estuarine/Marine	0.32	^81^
*Palaemonetes pugio*	Grass shrimp	Estuarine/Marine	0.32 (adult)0.68 (larvae)	^37^
*Macrobrachium rosenbergii*	Giant river prawn	Brackish water/Freshwater	0.98	^34^
*Farfantepenaeus aztecus*	Brown shrimp	Estuarine/Marine	1.3	Current study
*Macrobrachium nipponensis*	Oriental river shrimp	Freshwater	4.32	^34^
*Amphiascus tenuiremis*	Copepod	Estuarine/Marine	6.8	^82^
*Diaptomus castor*	Copepod	Freshwater	7.9 *	^20^
*Eriocheir sinensis*	Chinese mitten crab	Estuarine/Freshwater	8.56	^34^
*Procambarus clarkii*	Red swamp crayfish	Freshwater	14.3	^35^
*Ceriodaphnia dubia*	Water flea	Freshwater	17.7*	^83^
*Procambarus zonangulus*	White river crayfish	Freshwater	19.5	^35^
*Procambarus clarkii*	Red swamp crayfish	Freshwater	163.5	^81^
*Daphnia magna*	Water flea	Freshwater	190.0*	^84^
*Acanthocyclops robustus*	Copepod	Freshwater	194.2*	^20^

Values with star (*) indicate LC_50_ for48-h exposure. Table was modified from Chandler *et al*.^82^.

### Weight gain and growth rate

At the beginning of the experiment, there was no significant difference in the initial weight among the treatments; initial weight of shrimp ranged between 0.78 ± 0.08 g in the 1.0 µg/L treatment and 0.82 ± 0.08 g in the 6.4 µg/L treatment (Table 4).

**Table 4:** Initial weight (g), final weight (g), and % weight gain (mean ± standard deviation) of juvenile shrimp exposed to different concentrations of fipronil.

Fipronil concentrations (µg/L)	Initial weight (g)	Final weight (g)	Week of final weight measurement	% Weight gain
Control	0.80 ± 0.08 (n = 3) **a**	1.28 ± 0.08 (n = 3) **a**	4	60.19 ± 15.44 (n = 3) **a**
0.1	0.79 ± 0.05 (n = 3) **a**	0.71 (n = 1) **b**	3	− 8.97 (n = 1) **b**
1.0	0.78 ± 0.08 (n = 3) **a**	0.55 (n = 1) **b**	3	−21.42 (n = 1) **b**
3.0	0.82 ± 0.09(n = 3) **a**	0.81 ± 0.24 (n = 2) **b**	1	2.77 ± 19.64 (n = 2) **b**
6.4	0.82 ± 0.08 (n = 3) **a**	0.66 ± 0.17 (n = 3) **b**	1	−16.87 ± 29.57 (n = 3) **b**
10.0	0.81 ± 0.06(n = 3) **a**			

n = number of replicates in each treatment. All values were calculated based on the wet weight per individual shrimp. Means in columns not sharing the same letter are significantly different (P < 0.05).

**Figure 3:**
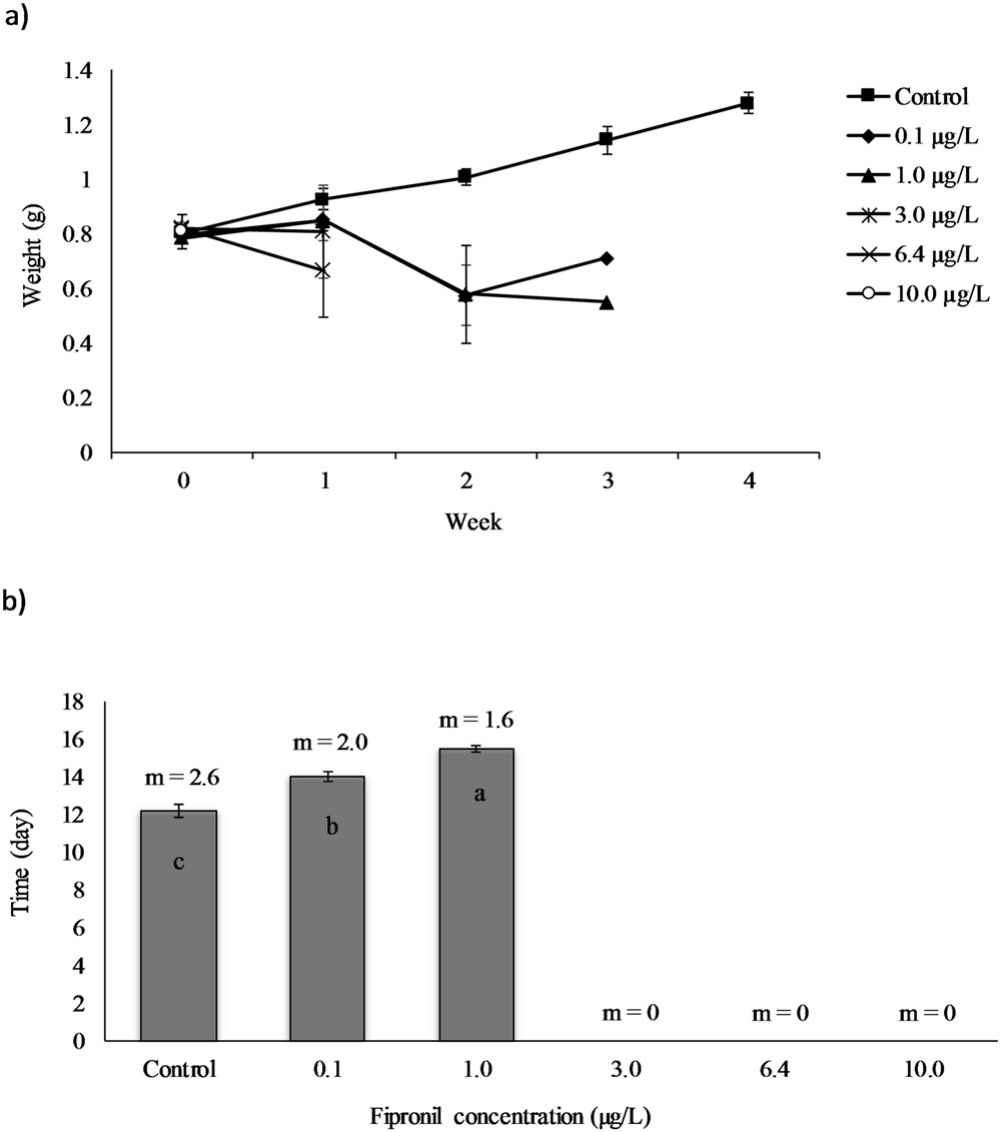
(**a**) Average weight (g wet weight per individual) of juvenile shrimp as a function of the duration of the exposure to fipronil. The horizontal axis represents the experiment period per week (week 0–week 4) while the vertical axis represents the average wet weight (g) per individual shrimp in each treatment. Error bars indicate the standard errors (n = 18). One-way Analysis of Variance (P < 0.05) showed that, after one week of fipronil exposure all treatments were significantly different from the control. (**b**) Inter-moult intervals of juvenile shrimp exposed to different concentrations of fipronil for 29 days. The horizontal axis represents the six fipronil concentrations (µg/L) including the control used during the experiment while the vertical axis represents time (per day) of the inter-moult intervals of juvenile shrimp. Error bars indicate the standard errors (n = 18). m is the average number of moults of individual shrimp in each treatment. Non- parametric Kruskal-Wallis test followed by the pairwise Wilcoxon rank sum test (P < 0.0001) showed that means in columns not sharing the same letter were significantly different.

The final weight in Table 4 was calculated for each treatment by taking the final weight measured before the death of all shrimp. However, the week of death of the last shrimp was different among the treatments. For example, the final weights of the 0.1 µg/L and 1.0 µg/L treatments were measured at the end of week 3; whereas, final weights of 3.0 µg/L and 6.4 µg/L treatments were measured at the end of week 1 of fipronil exposure because all shrimp in these treatments died before reaching week 2. However, final weight and percent weight gain clearly showed the effect of fipronil. In all treatments, a significant reduction in growth was observed after the first week of fipronil exposure (ANOVA, P < 0.05) (Table 4 and Fig. 3a).

The percent weight gain showed significant differences among all treatments (ranging from −21.42 g in the 1.0 µg/L treatment to 2.77 ± 19.64 g in the 3.0 µg/L treatment) and the control (60.19 ± 15.44 g) (ANOVA, P < 0.05). Weight loss occurred (−8.97, −21.42, and −16.87 ± 29.57 g) in three concentrations (0.1, 1.0, and 6.4 µg/L, respectively), indicating the final weight was less than the initial weight under all of these treatments. Final weight and percent weight gain in the 10.0 µg/L treatment were not measured because all individuals died during the first days of exposure before measuring the weight in week 1 (Table 4).

### Inter-moult interval

Control shrimps showed the highest number of moults per individual (m = 2.6); whereas, the number of moults decreased progressively with fipronil concentration. Inter-moult interval of the control treatment (12.2 ± 1.64 day) was significantly shorter than 0.1 μg/L treatment (14.0 ± 0.85 day) and the 1.0 μg/L treatment (15.5 ± 0.53 day) according to the Kruskal-Wallis rank sum test (P < 0.0001) (Fig. 3b).

We could not calculate the inter-moult intervals for shrimp in treatments of higher fipronil concentrations (3.0, 6.4, and 10.0 μg/L) because they died before they have two consecutive moults of any individual during the experiment.

### Behavioral and physical changes

Shrimp under high fipronil concentrations (3.0, 6.4, and 10.0 µg/L) showed behavioral changes after only one day. These changes were observed in the following order: (1) shrimp in these treatments started moving in circles with no control on their movements; (2) shrimp stopped moving in circles and sprawled on their sides or backs on the bottom of the aquarium with only their swimming legs moving in continuous involuntary movements; and (3) shrimp stopped moving their swimming legs and died. All of these abnormal swimming and feeding behaviors were recorded to compare them with shrimp in the control treatment. It is important to note that during all of these stages of abnormal behaviors, shrimp were not able to feed effectively. This was clearly observable under low fipronil concentrations (0.1 and 1.0 µg/L) in which shrimp survived for a longer period and exhibited the behavioral changes progressively and slowly.

**Figure 4:**
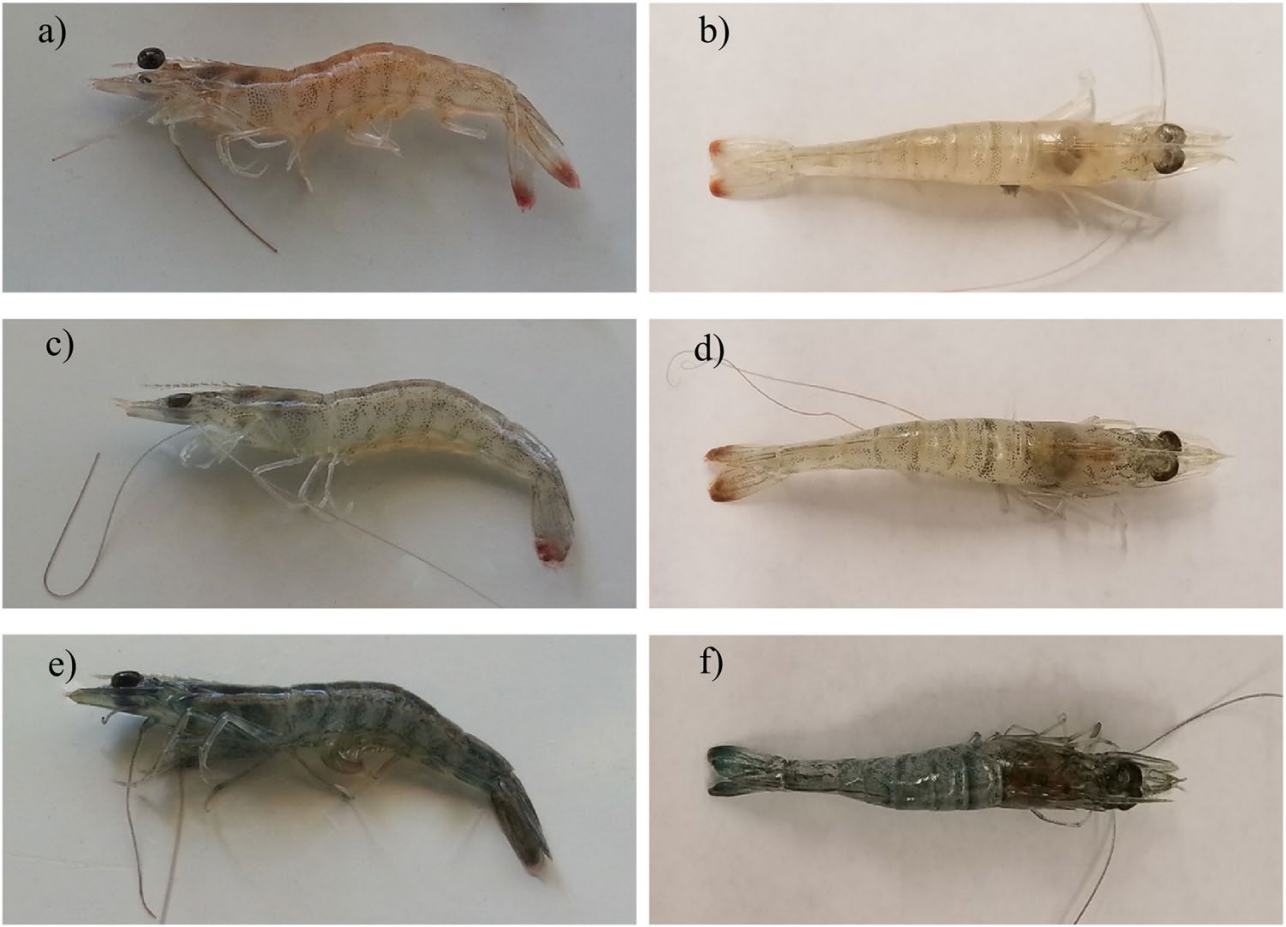
Body color of juvenile shrimp exposed to different concentrations of fipronil. Controls (**a**,**b**), 0.1 µg/L treatment (**c**,**d**), 1.0 µg/L treatment (**e**,**f**).

The visual examinations of the physical changes in shrimp bodies at the end of the experiment indicated a clear difference in their body color. Figure 4 shows shrimp body color gradient from bright color of shrimp in the control (0.0 µg/L) to gray and dark color of shrimp in the 0.1 µg/L and 1.0 µg/L treatments. This result indicates that fipronil affected shrimp body color in a concentration-dependent manner.

### Analysis of whole-body composition

**Figure 5:**
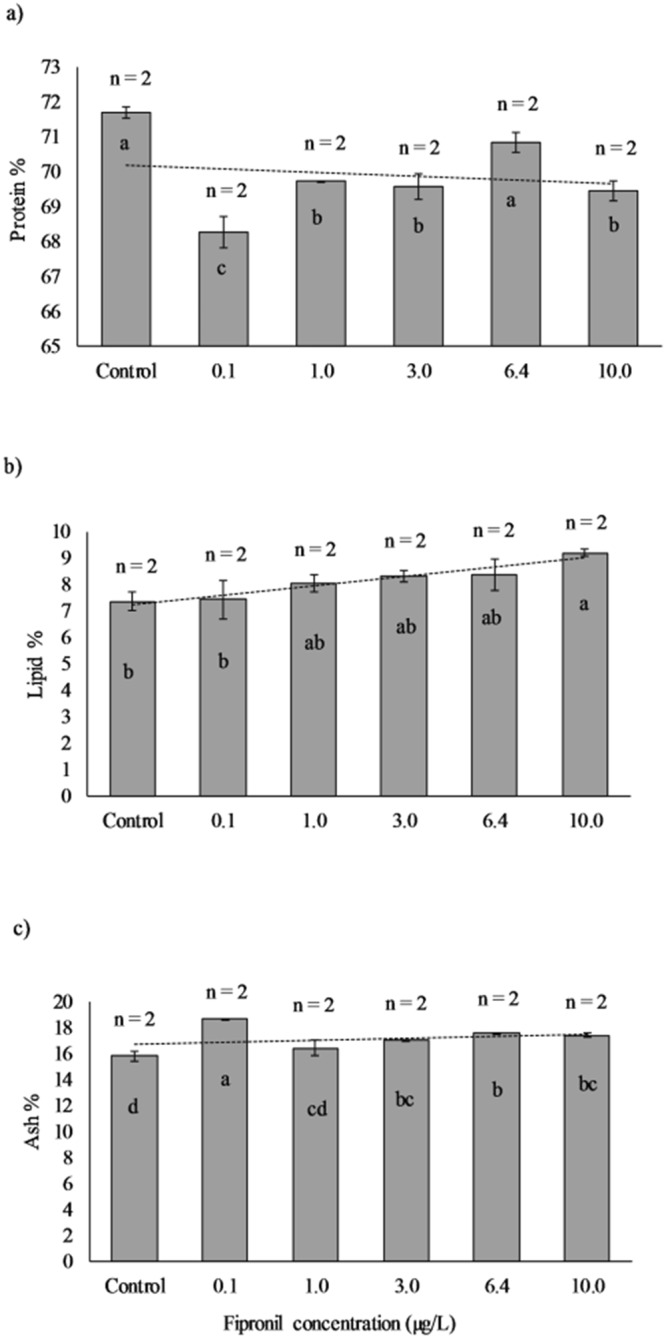
Analysis of body composition of juvenile shrimp under different fipronil concentrations. The horizontal axes in (6.a, 6.b, and 6.c) represent the six fipronil concentrations (µg/L) used during the experiment while the vertical axes represent the protein % (in 6.a), lipid % (in 6.b), and ash % (in 6.c) in bodies of juvenile shrimp measured at the end of the experiment. Dashed lines are fitted regression lines. Error bars indicate the standard errors (n = number of samples analyzed from each treatment). One-way Analysis of Variance (P < 0.05) showed that means in columns not sharing the same letter are significantly different.

Figure 5 shows the analysis of protein, lipid, and ash composition (dry basis) of juvenile shrimp in all treatments. Our results revealed that there were some significant differences (ANOVA, P < 0.05) among treatments in all components analyzed. There was an overall significant decrease (ANOVA, P < 0.05) in the percentage of body protein for all treatments compared to the control (0.0 µg/L) which showed the highest level of protein 71.69 ± 0.23%, although there was not a clear trend (Fig. 5a).

The ANOVA (P < 0.05) of lipid percentage indicated that there was no difference among treatments with the five lower concentrations, including the control. Similarly, there was no difference among treatments (ANOVA, P < 0.05) with the four higher concentrations (Fig. 5b). However, a linear regression analysis (P = 0.0017) indicated that lipid percentage increased significantly with increasing concentration of fipronil (see Supplementary Fig. S1).

For ash percentage, our analysis showed that the differences among groups in this case appear to be random and not associated with the insecticide exposure; although, control treatment had the lowest ash percentage (15.78 ± 0.53%) and differed significantly (ANOVA, P < 0.05) from most of the treatments (Fig. 5c).

## Discussion

Fipronil is known to cause lethal and sub-lethal effects on non-target invertebrates in both aquatic and terrestrial ecosystems^46^. However, studies are often conducted with a limited number of model organisms. Consequently, there is a gap of knowledge in the effects on a large number of non-target invertebrates, especially from coastal and marine ecosystems^47^. Fipronil desulfinyl (a photodegradation product of fipronil) was detected in the eggs of the Atlantic blue crab *Callinectes sapidus* off the coast of South Carolina (the Eastern coast of the United States), and it may be one of the causes of *C*. *sapidus* decline to the lowest historical levels over the past decade^33^. In Texas, fipronil has been reported in several recent studies conducted by the U.S. Geological Survey (USGS) and U.S. EPA in different cities including Houston-Galveston^48^, Austin^49^, San Antonio^50^, and College Station^51^, in concentrations ranging between 0.021 μg/L and 0.075 μg/L. All of these studies have reported the detection of fipronil and two or more of its degradation products (i.e., fipronil sulfide, fipronil sulfone, desulfinylfipronil, and desulfinylfipronil amide) in surface water and urban streams in levels exceeding the chronic level of the U.S. EPA aquatic life benchmark for invertebrates (0.01 μg/L). To the best of our knowledge, this study is the first to report the effects of fipronil on commercially and ecologically important penaeid shrimp *F*. *aztecus*.

All nominal fipronil concentrations tested in this study were within the range of concentrations found in the environment by other researchers in streams, rivers, and estuaries in the U.S. and other countries^22,30,52,53^ (see Supplementary Table S1). Our results showed fipronil caused significant lethal and sub-lethal effects on juvenile *F*. *aztecus*. Results also showed that survival of shrimp was concentration-dependent (Table 2 and Fig. 2). All individuals died during the 29 days of exposure under all the fipronil concentrations tested; whereas, no individual died in the control. The nominal 96-h LC_50_ of fipronil for juvenile *F*. *aztecus* was estimated at 1.3 μg/L. This result suggests *F*. *aztecus* have an intermediate sensitivity to fipronil among marine invertebrates, but they are far more sensitive than freshwater invertebrates studied so far (Table 3).

In our study, final weight and percent weight gain of shrimp showed significant differences (P < 0.05) between the control and all other concentrations (Table 4 and Fig. 3a). Growth reduction of aquatic arthropods under the exposures to toxicants also has been reported in other studies with sand shrimp *Metapenaeus ensis*^54^ and freshwater crayfish *Cherax quadricarinatus*^55^. A similar reduction in body growth of *F*. *aztecus* has been documented by Rozas *et al*.^56^, who found the reduction in the growth of juvenile *F*. *aztecus* and white shrimp *Litopenaeus setiferus* held for 7 days in field mesocosms contaminated with the nonlethal concentrations of petroleum hydrocarbons from an oil spill. On the contrary, Goff *et al*.^33^ found that juvenile blue crabs *Callinectes sapidus* exposed to different nominal concentrations of fipronil and fipronil desulfinyl resulted in significant increases in growth in all treatments compared to controls in a short-term (96-h) experiment.

There are several reasons that may explain the decrease in the growth of juvenile *F*. *aztecus* in our study. For instance, animals affected by environmental stressors, such as the chemical toxicants, utilize the energy in the detoxification processes, thus affecting the metabolism of protein and carbohydrate and eventually growth performance^55^. Shrimp derive energy more expeditiously from protein than from lipids and carbohydrate^57^; therefore, exposing *F*. *aztecus* to fipronil may have resulted in a reduced protein level in exposed shrimp compared with those in the control (Fig. 5a), which might have, in turn, reduced growth (Fig. 3a). On the other hand, fipronil is a phenylpyrazole insecticide, which acts by blocking the chloride channels, disrupting the central nervous system activity^13^, which may have inhibited feeding activity of juvenile *F*. *aztecus* under exposure.

Moulting is one of the important physiological processes for arthropods allowing them to grow normally^58,59^. Because moulting in crustaceans is mainly controlled by the interaction of moult-stimulating hormones (ecdysteroids), moult-inhibiting hormones (produced in the eyestalks), and nervous system secretions, endocrine disrupting chemicals, including fipronil^38^ in our study, are expected to have adverse effects on moulting^60^. In this study, fipronil affected *F*. *aztecus* moulting process in a concentration-dependent manner. Inter-moult intervals of shrimp under the control (12.2 ± 1.64 day) were significantly shorter (P < 0.0001) than those in other fipronil treatments (Fig. 3b). Increased inter-moult intervals suggest the development of shrimp is delayed by exposure to sub-lethal levels of fipronil in water.

Similar delay in moulting has been reported with other arthropods exposed to pesticides. Betancourt-Lozano *et al*.^61^ showed significant increase in inter-moult intervals of juvenile Pacific white shrimp *Litopenaeus vannamei* under the exposure to Tilt (a commercial formulation of the fungicide propiconazole). Snyder & Mulder^62^ showed delayed moulting of American lobster *Homarus americanus* larvae exposed to cyclodiene pesticide heptachlor. Baldwin *et al*.^63^ reported that juveniles and adults of the freshwater crustacean *Daphnia magna* exhibited reduced moulting frequency after they were chronically exposed to diethylstilbestrol (DES). Moreover, there are also reports of reduced moulting intervals, for example, with freshwater shrimp *Caridina nilotica* under exposure to the herbicide Roundup^®^^59^ and grass shrimp *P*. *pugio* under exposure to sodium pentachlorophenate and Aroclor 1242^64^. These studies suggest potentially complex mechanisms of pesticides affecting the moulting of arthropods.

Behavioral changes are often the first indication of the harmful impacts of pesticides on living organisms, and even at low doses of pesticides, long-term behavioral changes can be observed. This effect is magnified especially if the pesticide exposure occurred during the developmentally critical periods of the organism’s life^65^. In the present study, behavioral changes were observed under all fipronil concentrations compared with those under the control, starting from day 1 in the high concentration treatments and later in lower concentration treatments. Change in swimming (mobility) and feeding activities were the main observed changes. Similar results have been reported by other researchers. For example, Stratman *et al*.^27^ showed that the chironomid midge *Cricotopusle betis* Sublette exposed to fipronil exhibited abnormal behaviors, movement restriction, and feeding reduction. Overmyer *et al*.^66^ observed abnormal behavior and muscle control in the aquatic insect *Simulium vittatum* under all fipronil concentrations tested in the study.

Color changes were clearly observed in both the exoskeleton and abdominal muscle (Fig. 4). Because the body color of shrimp under 1.0 µg/L fipronil (Fig. 4e,f) were darker than those under 0.1 µg/L fipronil (Fig. 4c,d), which were, in turn, darker than that in the control (Fig. 4a,b), we concluded that the effect of fipronil on the color of juvenile *F*. *aztecus* was concentration-dependent. In crustaceans, and especially shrimp, many environmental factors are known to affect body color by affecting pigment dispersion (movement) within the chromatophores^67^. However, we note that the factors that are known to have an effect on body color of shrimp, such as temperature, light intensity, and background color, were carefully controlled in our study (Table 1). Body color in shrimp is often considered a sign of shrimp health, and consequently, influencing its commercial value^68^; for the same reason, color change in crustaceans, which is a hormonally-regulated process, can be used as a biomarker of environmental health^64^.

Some changes in body chemical compositions were observed under the exposure to fipronil in our study. A linear regression analysis showed a significant increase (P = 0.0017) in lipid percentage with fipronil concentration (see Supplementary Fig. S1). Similar results were found with juvenile mud crab *Rhithropanopeus harrisii* exposed to the insecticide fenoxycarb^69^, Pacific white shrimp *L*.*vannamei* exposed to oxytetracycline (OTC)^70^, and freshwater crayfish *C*. *quadricarinatus* exposed to glyphosate acid and polyoxyethylenamine (POEA)^55^, and freshwater amphipod *Gammarus pulex* exposed to the insecticide imidacloprid^71^. Protein percentage may also have been affected by fipronil (Fig. 5a). Although the protein percentage under the control (71.69 ± 0.23%) did not differ with those in the 6.4 µg/L treatment (70.84 ± 0.41%), it may be because of the fact that those in higher concentrations died early in the first days of the experiment, and they did not have enough time to exhibit a measurable reduction in protein percentage. Both protein and lipid metabolism are potentially affected by detoxification process^55^. If so, we would expect the effects to be concentration-dependent. However, they are also affected by the duration of exposure and feeding rate, which are also affected by toxicants. Further studies are needed for determining the existence of effects of fipronil on body chemical composition as well as potential mechanisms.

## Conclusion

Results of the present study revealed that the insecticide fipronil under concentrations found in the environment caused both lethal (acute) and sub-lethal (chronic) effects on *F*. *aztecus* juveniles. In particular, we found the effects on shrimp survival, growth (weight and moulting), swimming (mobility), feeding behavior, exterior appearance (body color), and body composition. Because of the detection of fipronil in estuarine waters, expected increased use of fipronil in areas adjacent to estuarine and coastal areas in the U.S.A and other countries, degradation of fipronil in the environment to multiple metabolites that pose equal or greater toxicity than fipronil itself, the high possibility of fipronil bioaccumulation in non-target organisms, and high commercial and ecological value of penaeid shrimp and their sensitivity to fipronil, we recommend the following: (1) monitoring fipronil concentration around the coastal regions in and out of the U.S.A., (2) trying to limit the use of fipronil during the peak periods of shrimp migration to estuaries, (3) investigating the effects of fipronil on different penaeid species in other countries that are using fipronil, and (4) conducting further studies of the effects of fipronil and its major metabolites on other non-target organisms using concentrations below chronic levels established by the U.S. EPA for marine invertebrates.

## Materials and Methods

### Test organisms and acclimation to laboratory conditions

Juvenile brown shrimp *F*. *aztecus* (weight 0.80 ± 0.06 g, total length 5.0 ± 0.67 cm) were collected from Gangs Bayou, Sportsman Road (N 29.25549; W 94.91575) in Galveston Bay, Texas, using a 3-m bag seine (0.6 cm mesh size) on May 6, 2016. Shrimp were transported in 45-liter coolers equipped with air pumps to the laboratory in Texas A&M University, College Station, Texas. After equilibrating water temperature of the transportation coolers with laboratory temperature over approximately 5 hours (see Supplementary Fig. S2a), active shrimp were selected and moved to 53-liter plastic tanks filled with aerated artificial brackish water, which was prepared with dechlorinated tap water and Instant Ocean^®^ Sea Salt (see Supplementary Fig. S2b).

Shrimp were acclimated to laboratory conditions in the tanks for 10 days at temperature, 19.93 ± 0.15 °C; salinity, 15.75 ± 0.16‰; pH, 8.14 ± 0.18; and photoperiod, 12 hour: 12 hour light: dark cycle (see Supplementary Fig. S2a). During the acclimation period, shrimp were fed on API^®^ Bottom Feeder Shrimp Pellets, which fit the nutritional requirements of shrimp^72^, twice a day. The acclimation tanks were cleaned daily to remove feces and uneaten food and approximately 30–40% of water was changed with newly prepared brackish water. At the end of the acclimation period, shrimp were moved to test aquariums to begin the experiment.

### Experimental design and water quality parameters

The experiment lasted 29 days from May 17, 2016 to June 14, 2016. The system consisted of 18 glass aquariums (six treatments x three replicates) of 9.5 liter (30.7 × 15.4 × 20.5 cm) (see Supplementary Fig. S2c), one aquarium was treated as one replicate. An aquarium was filled with 7 liters of test solution, equipped with air pumps (Topfin^®^ AIR-8000), and covered with a glass lid to prevent shrimp from escaping. Each aquarium was divided equally into six cells (see Supplementary Fig. S2d), and one individual was assigned to each cell to prevent cannibalism among shrimp and to follow moulting of each shrimp individually^73^. The divider was made of a polypropylene plate and fiberglass screen (see Supplementary Fig. S2d); both are commonly used for aquaculture purposes. The screen maintained the flow of water, which distributed dissolved oxygen among the cells. Additionally, aquariums were covered from all sides with aluminum foil sheets to minimize the degradation of fipronil due to light exposure during daytime (see Supplementary Fig. S2c). The aquariums were placed randomly in three rows. During the experiment, shrimp were fed twice daily. Food amount was adjusted according to the body weight, which was measured weekly, based on the published feeding tables for shrimp^72^. Dissolved oxygen concentration (mg/L), salinity (‰), temperature (°C), and pH were measured every other day using YSI^®^ Professional Plus Multi-parameter Meter.

### Insecticide, concentrations and test solutions

Fipronil (5-amino-1-[2, 6-dichloro4-4(trifluoromethyl) phenyl]-4[(trifluoromethyl) sulfinyl]-1H-pyrazole-3-carbonitrile), CAS number 120068-37-3 and purity limit ≥97% (HPLC), was purchased from Fisher Scientific Co. L.L.C., PA, US. Six nominal concentrations, including the control, were used for this experiment: 0.0, 0.1, 1.0, 3.0, 6.4, and 10.0 µg/L. These concentrations were selected based on those previously observed by other researchers in the environment^22,28,30,52^ (see Supplementary Table S1). Each treatment (concentration) was conducted in triplicate.

The nominal experimental solutions were prepared by making a 1 liter of highly homogenized 100 mg/L fipronil suspension; this suspension was made by mixing 0.1 g of fipronil powder in 1 liter of artificial brackish water using a magnetic stirrer. Then, all of the nominal experimental concentrations (0.1, 1.0, 3.0, 6.4, and 10.0 µg/L) were prepared by diluting specific quantities of 100 mg/L fipronil suspension with artificial brackish water. For example, to prepare 0.1 µg/L fipronil solution, we took 10 ml of 100 mg/L fipronil suspension and mixed it with 990 ml of prepared water to create 1 mg/L fipronil solution, and then, we took 2.1 ml of 1 mg/L fipronil solution and mixed it with 21 liters of prepared water. Dilutions of all nominal experimental concentrations are shown in Supplementary Table S2. For each nominal concentration, 21 liters of fipronil solution was created for three aquariums (replicates). To maintain the fipronil concentrations under all treatments during the experiment, 100% of test solutions were replaced every two days.

### Experimental measurements

All assays were conducted using the static-renewal method and according to the guidelines of the U.S. EPA^74^. The number of shrimp in each replicate and the number of replicates were determined referring to previous studies^34,73^.

### Survival, median lethal time (LT_50_), and acute toxicity test (LC_50_)

Survivorship of shrimp was measured by monitoring shrimp movements in the aquariums during feeding periods. Dead shrimp were removed, counted, and weighed. The weight of dead shrimp was used to adjust food amounts for remaining live shrimp. Shrimp were considered dead if they lay down on their side or back with no noticeable movement and they did not make any response (such as jumping, moving their legs, or flipping their tails) after taking them out of water. The dead individuals were placed in a freezer for later body chemical composition analysis. We used survivorship data to estimate the median lethal time (LT_50_) and also the acute toxicity of fipronil (LC_50_) on shrimp under 96-h of exposure.

### Weight gain and growth rate

Shrimp were weighed every week to observe the effect of fipronil concentrations as well as to adjust the amount of food. Shrimp were weighed individually after gently removing water with paper towel and placed in a beaker with known amount of brackish water. The weekly weight gain of shrimp was calculated using equation (1):

1 $$ \% \,\mathrm{Weight}\,{\rm{gain}}=({\rm{Final}}\,{\rm{weight}}-{\rm{Initial}}\,{\rm{weight}})/({\rm{Initial}}\,{\rm{weight}})\times 100$$

### Inter-moult interval

We calculated the inter-moult interval of shrimp under each concentration by counting the number of days between each two consecutive moults of the same individuals. This was possible because we isolated juvenile shrimp in cells (within the same aquarium) and covered the aquarium with a glass lid to prevent the movement of individuals among cells. Then, the date of moulting of each individual was recorded.

### Behavioral and physical changes

At each feeding time (morning and afternoon) and also at night, any abnormalities in shrimp activities as well as any changes in physical appearance compared with shrimp in the control were noted and recorded on video.

### Analysis of whole-body composition

At the end of the experiments, live shrimp were collected, euthanized by freezing them, and kept in freezer (at −18 °C). Eighteen individuals under each treatment was combined to create two samples. For each sample, dry matter of whole body of shrimp was measured first by accurately weighing 2.0 g of shrimp in a pre-weighed porcelain crucible, placing the samples in an oven at 135 °C for 3 hours^75^, and weighing them again. Then, porcelain lab mortar and pestle were used to prepare a highly homogenized shrimp powder to be used in subsequent analyses. The crude protein content of shrimp body was determined through Dumas protocol using a LECO protein analyzer to measure total nitrogen as described in^76^. Lipids were estimated using chloroform/methanol 2:1 extraction method^77^. Ash was determined by placing dry matter samples in a muffle furnace at 550 °C for 3 hours^75^.

### Statistical analysis

One-way Analysis of Variance (ANOVA) and linear regression were used to test for the significant differences among all treatments compared to the control. In some measurements such as the survivorship and inter-moult interval of shrimp, the data were not normally distributed, and non- parametric tests were used. Kaplan–Meier estimator was conducted to estimate shrimp survivorship followed by the non-parametric Log-Rank test to compare the survival distribution among treatments. Probit analysis described by Finney^78^ was used to calculate the LC_50_, using log concentration as dependent variable and probit as independent variable, then we used the parametric bootstrap method to calculate the 95% confidence intervals of the LC_50_ toxicity test^78^ (see Supplementary Fig. S3). Non- parametric Kruskal-Wallis test followed by the pairwise Wilcoxon rank sum test were used to test for differences among the means of treatments of the inter-moult intervals. All of these statistical analyses were conducted at significance level α = 0.05 using JMP^®^ Pro 2016^79^ (ANOVA, Kaplan–Meier, and Kruskal-Wallis tests), Matlab R2017a^80^ (LC_50_ calculations), and Microsoft Excel 2016 (linear regression test, and to draw all figures).

### Data Availability

The datasets generated during and/or analyzed during the current study are available from the corresponding author on reasonable request.

## Electronic supplementary material

Supplementary Information
