# Supplementary material for: Lethal and sub-lethal effects of the insecticide fipronil on juvenile brown shrimp Farfantepenaeus aztecus
Source: Sci Rep. 2018 Jul 17;8:10769. doi: 10.1038/s41598-018-29104-3 (PMC6050305; doi:10.1038/s41598-018-29104-3)
Supplement: Supplementary file 1 — Supplementary Information [file 41598_2018_29104_MOESM1_ESM.docx]

Lethal and sub-lethal effects of the insecticide fipronil on juvenile brown shrimp *Farfantepenaeus aztecus*

Ali Abdulameer Al-Badran^1,*^, Masami Fujiwara^1^, Delbert M. Gatlin III^1^, Miguel Mora^1^

1. Department of Wildlife and Fisheries Sciences
Texas A&M University
College Station, TX 77843-2258, U.S.A.


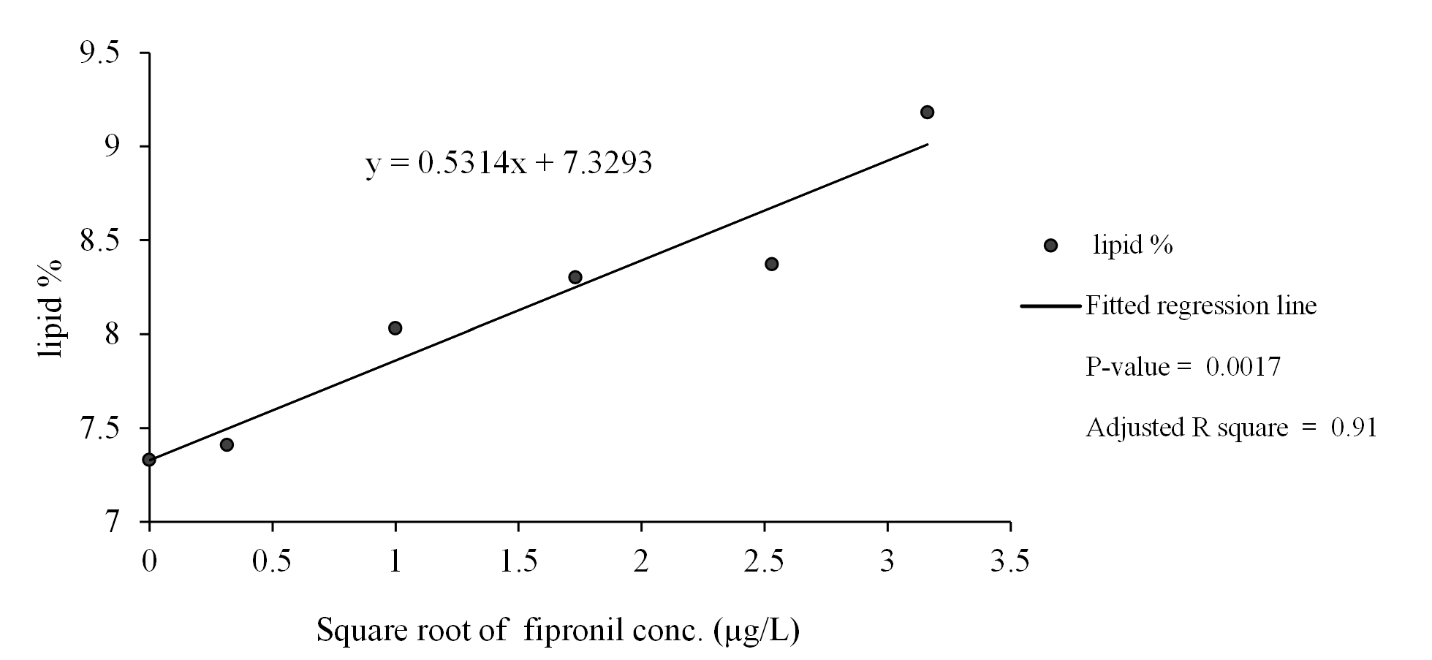


**Supplementary Figure S1.** Linear regression of lipids % of shrimp bodies measured under different concentrations of fipronil. The horizontal axis represents the square root of fipronil concentrations (µg/L), and the vertical axis represents the lipid % in bodies of juvenile shrimp measured at the end of the experiment (n = 2 samples analyzed from each treatment). Linear regression analysis (P = 0.0017) indicated that lipid % increased significantly with increasing concentration of fipronil.


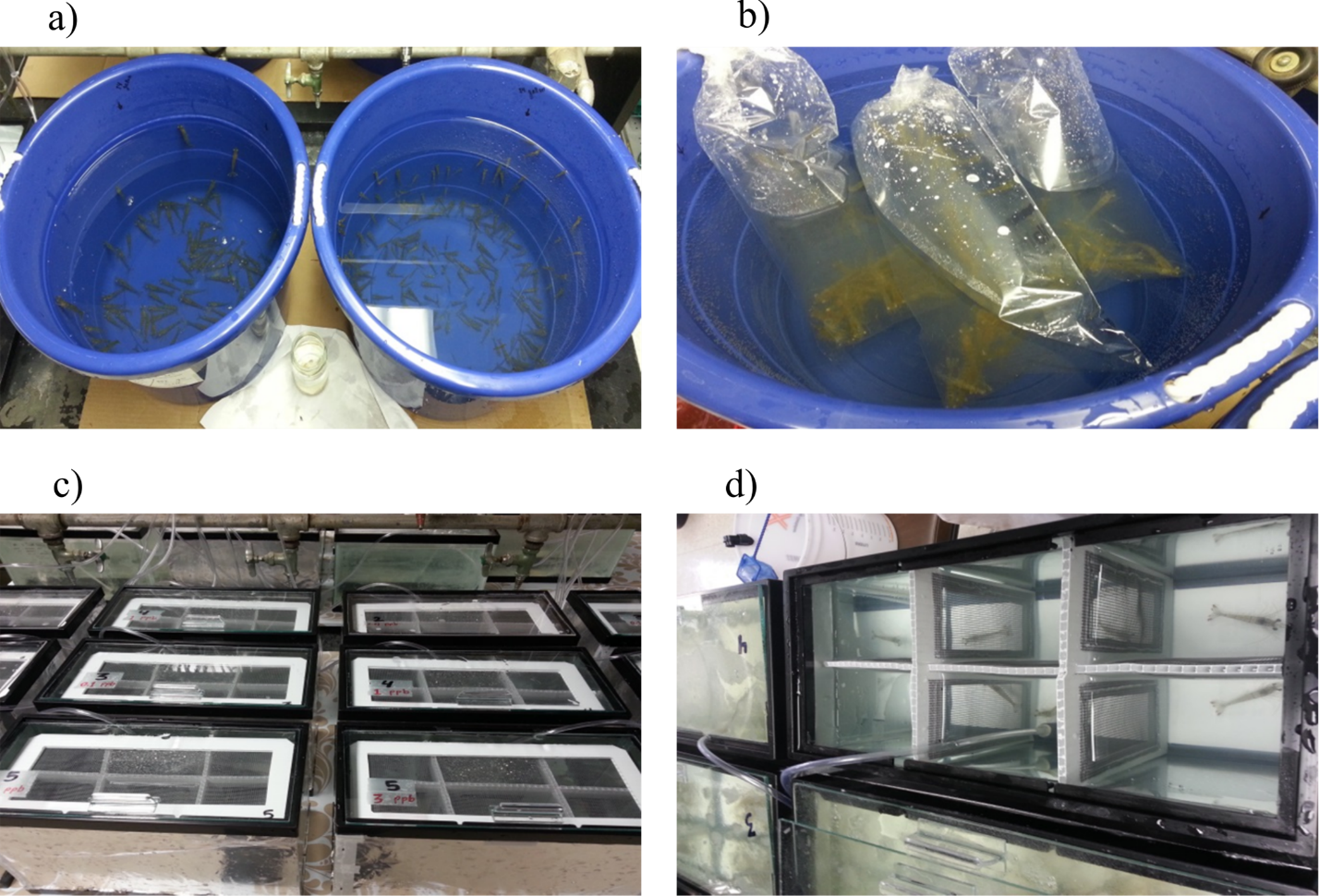


**Supplementary Figure S2.** Acclimation tanks and experimental system used for juvenile *F. aztecus* laboratory experiments: (**a**) large tanks used for water temperature equilibration and shrimp acclimation before starting the trials; (**b**) procedure used for moving shrimp to tanks of prepared brackish water for acclimation to laboratory conditions; (**c**) experimental system, glass aquariums covered with aluminum foil sheets and glass lids; (**d**) aquariums divided into six separate cells.


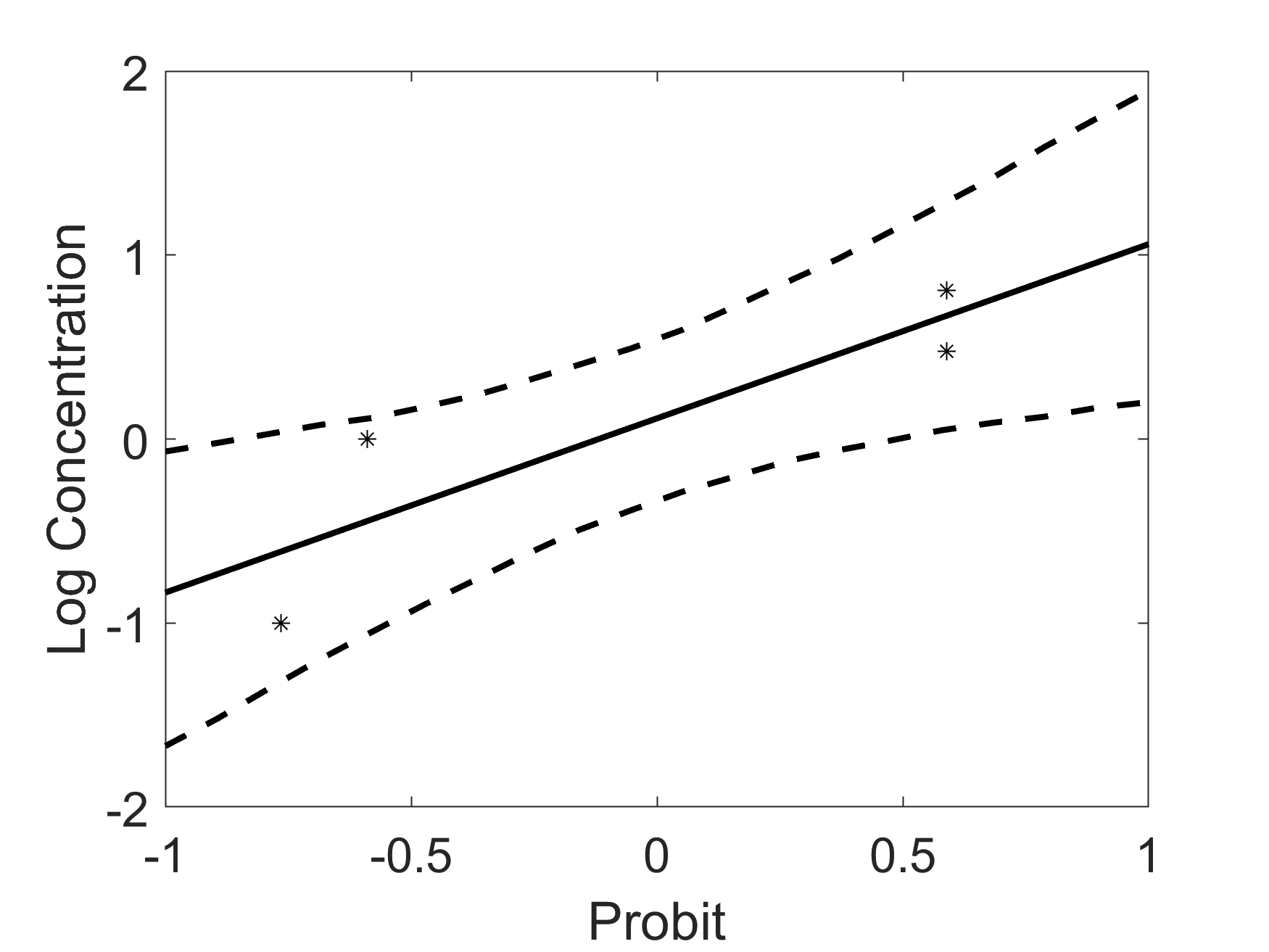


**Supplementary Figure S3.** Probit analysis used to calculate the 96-h LC_50_ of fipronil. The horizontal axis represents the probit as independent variable while the vertical axis represents the log concentration as dependent variables. Dashed lines show the 95% confidence intervals of the LC_50_ toxicity test calculated using the parametric bootstrap method.

| **Detected concentrations of fipronil in environment (µg/L)** | **Year of the survey** | **State/Country** | **Reference** |
| --- | --- | --- | --- |
| **0.09** ^a^-**10.004** | 2003-2012 | California, U.S.A | ^1^ |
| 0.004 -**6.41** | 2006 | Louisiana, U.S.A | ^2^ |
| 0.829 - 5.29 | 2000 | Louisiana, U.S.A | ^3^ |
| **1.0** | 2010 - 2011 | Tsukuba, Japan | ^4^ |
| 0.63 ^c^ | 2006 | Texas, U.S.A | ^5^ |
| 0.03 | 2004 - 2005 | Texas, U.S.A | ^6^ |
| 0.3 - 0.8 | 1999 - 2001 | Louisiana, U.S.A | ^7b^ |
| 0.007 - 6.0 | 1992 - 2001 | U.S.A | ^8^ |
| 0.0006 - 0.0086 | 2002 - 2004 | Florida, U.S.A | ^9^ |
| 0.0018 - 10.004 | 2006 - 2008 | California, U.S.A | ^10^ |
| 0.01 - 4.2 | 2007 - 2008 | California, U.S.A | ^11^ |
| 0.28 - 2.11 | 2008 - 2011 | California, U.S.A | ^12^ |
| 0.0145 - 0.0274 | 2014 | California, U.S.A | ^13^ |

**Supplementary Table S1.** Fipronil concentrations observed in the aquatic environment, year, and the place of the survey. ^a^ 0.09 µg/L was rounded to 0.1 µg/L during the experiment. ^b^ U.S. Geological Survey, Baton Rouge, LA, USA, unpublished data. ^c^ Value measured in µg/g OC (% of organic carbon in the sediment). Concentrations in bold are concentrations used in current study.

| Fipronil concentration  µg/L | Dilution steps | | |
| --- | --- | --- | --- |
|  | Step 1 | Step 2 | Step 3 |
|  | 100 mg/L Fipronil  suspension | 1 mg/L Fipronil  solution |  |
| 0.1 | Mix 0.1 g of fipronil powder in 1000 ml of brackish water | Mix 10 ml of 100 mg/L fipronil suspension in 990 ml of brackish water | Mix 2.1 ml of 1 mg/L fipronil solution in (21,000 ml – 2.1 ml) of water |
| 1.0 |  |  | Mix 21 ml of 1 mg/L fipronil solution in (21,000 ml – 21 ml) of water |
| 3.0 |  |  | Mix 63 ml of 1 mg/L fipronil solution in (21,000 ml – 63 ml) of water |
| 6.4 |  |  | Mix 134.4 ml of 1 mg/L fipronil solution in (21,000 ml – 134.4 ml) of water |
| 10.0 |  |  | Mix 210 ml of 1 mg/L fipronil solution in (21,000 ml – 210 ml) of water |

**Supplementary Table S2.** Dilutions of all nominal fipronil concentrations used in the experiment. For steps 1 and 2, we used 2 flasks of 1000 ml and magnetic stirrer to assure the fully homogenize of the experimental solutions.

References

1 Ruby, A. Review of pyrethroid, fipronil and toxicity monitoring data from california urban watersheds. *California Stormwater Quality Association (CASQA)*, 90 p. (2013).

2 Mize, S. V., Porter, S. D. & Demcheck, D. K. Influence of fipronil compounds and rice-cultivation land-use intensity on macroinvertebrate communities in streams of southwestern Louisiana, USA. *Environ. Pollut.* **152**, 491-503 (2008).

3 USGS. Fipronil and degradation products in the rice-producing areas of the mermentau river basin, Louisiana, February–September 2000. Fact Sheet FS-010-03. *U.S. Geological Survey (USGS)*, 6 p (2003).

4 Hayasaka, D. *et al.* Cumulative ecological impacts of two successive annual treatments of imidacloprid and fipronil on aquatic communities of paddy mesocosms. *Ecotoxicol. Environ. Saf.* **80**, 355-362 (2012).

5 Hintzen, E. P., Lydy, M. J. & Belden, J. B. Occurrence and potential toxicity of pyrethroids and other insecticides in bed sediments of urban streams in central Texas. *Environ. Pollut.* **157**, 110-116 (2009).

6 Sneck-Fahrer, D. A. & East, J. W. Water-quality, sediment-quality, stream-habitat, and biological data for Mustang Bayou near Houston, Texas, 2004–05. *U.S. Geological Survey Data Series 263*, 90 p. (2007).

7 Chandler, G. T. *et al.* Fipronil effects on estuarine copepod (Amphiascus tenuiremis) development, fertility, and reproduction: a rapid life-cycle assay in 96-well microplate format. *Environ. Toxicol. Chem.* **23**, 117–124 (2004).

8 Gilliom, R. J. *et al.* The Quality of Our Nation’s Waters—Pesticides in the Nation’s Streams and Ground Water, 1992–2001. U.S. Geological Survey Circular 1291. 172 p. (2006).

9 Harman-Fetcho, J. A. *et al.* Pesticide Occurrence in Selected South Florida Canals and Biscayne Bay during High Agricultural Activity. *J. Agric. Food Chem.* **53**, 6040−6048 (2005).

10 Gan, J., Bondarenko, S., Oki, L., Haver, D. & Li, J. X. Occurrence of fipronil and its biologically active derivatives in urban residential runoff. *Environ. Sci. Technol.* **46**, 1489-1495 (2012).

11 Greenberg, L. *et al.* Impact of ant control technologies on insecticide runoff and efficacy. *Pest. Manag. Sci.* **66**, 980-987, doi:10.1002/ps.1970 (2010).

12 Ensminger, M., Budd, R., Kelley, K. C. & Goh, K. S. Pesticide occurrence and aquatic benchmark exceedances in urban surface waters and sediments in three urban areas of California, USA, 2008–2011. *Environ. Monit. Assess.* **185**, 3697–3710 (2013).

13 Weston, D. P., Chen, D. & Lydy, M. J. Stormwater-related transport of the insecticides bifenthrin, fipronil, imidacloprid, and chlorpyrifos into a tidal wetland, San Francisco Bay, California. *Sci. Total. Environ.* **527-528**, 18-25, doi:10.1016/j.scitotenv.2015.04.095 (2015).
